# The Signal Transducer and Activator of Transcription 1 (STAT1) Inhibits Mitochondrial Biogenesis in Liver and Fatty Acid Oxidation in Adipocytes

**DOI:** 10.1371/journal.pone.0144444

**Published:** 2015-12-21

**Authors:** Jennifer D. Sisler, Magdalena Morgan, Vidisha Raje, Rebecca C. Grande, Marta Derecka, Jeremy Meier, Marc Cantwell, Karol Szczepanek, William J. Korzun, Edward J. Lesnefsky, Thurl E. Harris, Colleen M. Croniger, Andrew C. Larner

**Affiliations:** 1 Department of Biochemistry and Molecular Biology, and Massey Cancer Center, Virginia Commonwealth University, Richmond, VA, 23298, United States of America; 2 Department of Pharmacology, University of Virginia School of Medicine, Charlottesville, VA, 22908, United States of America; 3 Medical Service, McGuire Department of Veterans Affairs Medical Center, Richmond, VA, 23249, United States of America; 4 Department of Clinical Chemistry, Virginia Commonwealth University, Richmond, VA, 23298, United States of America; 5 Department of Nutrition, Case Western University School of Medicine, Cleveland, OH, 44106, United States of America; 6 Department of Internal Medicine, Division of Cardiology, and Pauley Heart Center, Virginia Commonwealth University, Richmond, VA, 23298, United States of America; East Tennessee State University, UNITED STATES

## Abstract

The transcription factor STAT1 plays a central role in orchestrating responses to various pathogens by activating the transcription of nuclear-encoded genes that mediate the antiviral, the antigrowth, and immune surveillance effects of interferons and other cytokines. In addition to regulating gene expression, we report that *STAT1* ^*-/-*^ mice display increased energy expenditure and paradoxically decreased release of triglycerides from white adipose tissue (WAT). Liver mitochondria from *STAT1* ^*-/-*^ mice show both defects in coupling of the electron transport chain (ETC) and increased numbers of mitochondria. Consistent with elevated numbers of mitochondria, *STAT1* ^*-/-*^ mice expressed increased amounts of PGC1α, a master regulator of mitochondrial biogenesis. STAT1 binds to the PGC1α promoter in fed mice but not in fasted animals, suggesting that STAT1 inhibited transcription of PGC1α. Since *STAT1* ^*-/-*^ mice utilized more lipids we examined white adipose tissue (WAT) stores. Contrary to expectations, fasted *STAT1* ^*-/-*^ mice did not lose lipid from WAT. β-adrenergic stimulation of glycerol release from isolated *STAT1* ^*-/-*^ WAT was decreased, while activation of hormone sensitive lipase was not changed. These findings suggest that *STAT1* ^*-/-*^ adipose tissue does not release glycerol and that free fatty acids (FFA) re-esterify back to triglycerides, thus maintaining fat mass in fasted *STAT1* ^*-/-*^ mice.

## Introduction

The classic JAK/STAT pathway controls cellular responses to cytokines and growth factors by regulating the expression of nuclear-encoded early response genes [1]. Cytokines binding to their cell surface receptors trigger the activation of one or several JAK tyrosine kinases, which phosphorylate the cytoplasmic domains of the receptor. Phosphorylated residues provide docking sites for the SH2 domains of STATs, allowing for their tyrosine phosphorylation by the JAK proteins. Phosphorylated STATs form homodimers or heterodimers, translocate to the nucleus, and bind to the promoters of cytokine-stimulated early response genes.

Although in the majority of cases, STAT1 and other STATs must be tyrosine phosphorylated to activate gene expression, reports indicate that there are sets of genes regulated by STAT1, STAT3 and other STATs that do not require these transcription factors to be phosphorylated [2, 3]. Unphosphorylated STAT1 regulates the expression of caspases [4], as well as proteins involved in glycolysis/gluconeogenesis, the tricarboxylic acid (TCA) cycle and oxidative phosphorylation [5]. The later studies were performed using squamous carcinoma cells where the expression of STAT1 was ablated using STAT1 shRNA [5]. Transcriptional profiling of these transformed cells in the absence and presence of STAT1 expression suggested that there were modest changes in levels of RNAs involved in glycolysis/gluconeogenesis and oxidative phosphorylation. The physiological consequences in these metabolic shifs was not examined.

There are many reports examining the pathophysiology of *STAT1* ^*+/+*^ and *STAT1* ^*-/-*^ mice, primarily in the context of immune responses, which involve cytokine activation of this transcription factor. Although there are several studies indicating that STAT3 can directly or indirectly affect cellular metabolism, in vivo [6, 7] to our knowledge, there have been no reports of alterations in metabolism in *STAT1* ^*-/-*^ mice under homeostatic conditions where STAT1 is not activated by tyrosine phosphorylation. We initiated these studies to examine whether STAT1 plays a role in controlling energy balance. To address this issue we used *STAT1* ^*-/-*^ mice, which allowed us to examine STAT1-mediated changes in metabolism *in vivo*. These studies highlight a previously unknown role of STAT1 in the regulation of triglyceride turnover, mitochondrial biogenesis, and energy utilization.

## Materials and Methods

### Materials

STAT1 monoclonal antibody was purchased from BD Transduction. Antibodies recognizing Tyrosine 701 Phosphorylated STAT1, HSL, Phospho-specific S563HSL that recognizes at the cAMP-dependent kinase site and IgG were all purchased from Cell Signaling. Tubulin antibody was purchased from Sigma.

### Mice

*STAT1* ^*-/-*^ and *STAT1* ^*+/+*^ mice (129/SV background) were purchased from Taconic Labs and housed in the central animal research facility of Virginia Commonwealth University School of Medicine. The protocol for mice has been approved per Institutional Animal Care and Use Committee (IACUC, protocol AM10091) regulations. All experiments were conducted with male mice 6–12 weeks of age. Every effort has been made to minimize discomfort and pain to the extent possible within the context of the proposed studies. Mice were sacrificed by CO_2_ inhalation and cervical dislocation. Animals were not kept alive for any significant time beyond that required for the studies.

### Biochemical analysis

We generated plasma using Microtainer plasma separator tubes (Becton Dickinson). Veterinary Diagnostic Services (Marshfield Laboratories) and measured the levels of cholesterol, β-hydroxybutyrate, FFA, and triglycerides by an automated analyzer (Roche Modular Autoanalyzer). We assayed liver triglycerides using the triglyceride GPO reagent as previously described [8].

### Body composition

*STAT1* ^*+/+*^ and *STAT1* ^*-/-*^ mice were fasted and then sacrificed. Animal carcasses were shipped to the Mouse Metabolic Phenotyping Center at the University of Cincinnati for body composition analysis. The body composition of fasted *STAT1* ^*+/+*^ and *STAT1* ^*-/-*^ mice was determined using quantitative magnetic resonance [9].

### Food intake

Ten week old *STAT1* ^+/+^ (n = 5) and *STAT1* ^*-/-*^ (n = 6) mice were separated into individual cages for 1 week prior to being used for experiments. A pre-weighed amount of 2919 mouse chow was added to each hopper, and then reweighed 24 hours later.

### Measurement of energy expenditure by indirect calorimetry

Metabolic rates were measured by indirect calorimetry in mice using an 8-chamber open-circuit Oxymax system (CLAMS, Columbus Instruments, Columbus, OH) at the Mouse Metabolic Phenotyping Center (MMPC) at Case Western Reserve University. Briefly, mice were acclimated to the experimental room for 1 week prior to the experiment. The mice were individually housed in acrylic calorimeter chambers through which air with a known O_2_ concentration is passed at a constant flow rate. The system automatically withdrew gas samples from each chamber hourly for 24 h. The system then calculated the volumes of O_2_ consumed (VO_2_) and CO_2_ generated (VCO_2_) by each mouse in 1 h. The RQ, which is the ratio of VCO_2_ to VO_2_, is calculated. Heat or energy expenditures were measured in both light and dark cycles and in both fed and fasting conditions and are represented as Kcal/g/day. Mice were maintained at 25°C and had free access to water in all conditions.

#### Measurement of triglyceride concentration and synthesis by stable isotopes

MMPC measured triglyceride content and newly synthesized triglyceride levels. Mice were trained to eat 10:00AM-12:00AM for one week prior to the experiment. On the day of the experiment to enrich body water to ~2% ^2^H, mice were injected IP with labeled water (^2^H_2_O) (20 mL/g body weight in 9 g/L NaCl in 99% atomic percentage excess ^2^H_2_O) and returned to their cages. Five hours later the mice were sacrificed and blood and tissue samples were collected and flash-frozen in liquid nitrogen. The samples were stored at 80°C until analysis. Triglyceride concentrations and de novo lipogenesis were analyzed as previously described [10]. Briefly, triglyceride from tissues was isolated and labeled glycerol and palmitate were analyzed after derivatization by MS. The ^2^H label on triglyceride covalently linked to glycerol measures the amount of newly synthesized triglyceride, whereas the ^2^H label in triglyceride covalently attached to palmitate indicates the amount of new palmitate. Calculations were performed as previously described [10].

### WAT analysis in fasted mice

8–10 week old male *STAT1* ^*+/+*^ and *STAT1* ^*-/-*^ mice were fasted for 24 h and the subcutaneous and gonadal fat pads were removed from the animals and weighed. Samples were averaged and compared between *STAT1* ^*+/+*^ and *STAT1* ^*-/-*^ mice.

### Oil Red O staining

Livers were harvested from *STAT1* ^*+/+*^ and *STAT1* ^*-/-*^ mice under basal and fasting conditions. Livers were rinsed in 1xPBS and then snap frozen in liquid nitrogen. The samples were fixed in 4% formaldehyde. Samples were then washed (while shaking) three times for 10 minutes each with 1xPBS. Samples were washed in 60% isopropanol for 30 seconds and were stained with Oil Red O (Sigma) for 15 minutes. Samples were then washed again in 60% isopropanol for 30 seconds and washed three times for 10 minutes each with 1xPBS. The samples were imaged at 10x and 40x.

### Primary adipocyte isolation

Primary adipocytes were isolated from subcutaneous white adipose tissue from *STAT1* ^*+/+*^ and *STAT1* ^*-/-*^ mice [11]. Harvested fat was minced into small pieces and incubated with Low Phosphate Buffer (LPB) (145mM NaCl, 5.4mM KCl, 1.4mM CaCl_2_, 1.4mM MgSO_4_, 0.2mM NaH_2_PO_4_, 5mM Glucose, 10mM HEPES) containing 2mg/mL collagenase (Worthington, Lakewood, NJ) in a 37°C shaker for 60 minutes. Digested fat was passed through 250 micron mesh (Fisher ThemoScientific, Pittsburgh, PA) to create a single cell suspension of the adipocytes and then washed with 10mL LPB containing 2.5% Insulin-free BSA (MP Biomedicals, Santa Ana, CA) and 200nM adenosine. Samples were centrifuged at 1000 RPM for 1 minute at room temperature. The supernatant was removed using a 10mL syringe. The adipocytes were washed twice with 10mL of LPB containing 0.5% Insulin-free BSA and centrifuged at 1000 RPM for 1 minute at room temperature between each wash. For the last wash, 10mL LPB was added and adipocytes were centrifuged at 1000 RPM for 1 minute at room temperature. The aqueous layer was removed and adipocytes were incubated at 37°C with 10μM isoproterenol or 10μM Forskolin for 60 minutes. Lyspolysis was analyzed using the free glycerol release kit from Sigma.

### RNA extraction and real-time PCR

Total RNA was isolated with TRI Reagent (Molecular Research, Cincinnati, OH), according to the manufacturer’s instructions. All the samples were assayed in duplicate and analyzed using a CFX96 Real-Time PCR Detection System (Bio-Rad, Hercules, CA). Table 1 contains a list of the primer sequences.

**Table 1:** Primer sequences used for qRT-PCR.

Primer Name	Sequence
PGC1α	F 5’-CCCTGCCATTGTTAAGACC-3’
	R 5’-TGCTGCTGTTCCTGTTTTC-3’
ND4	F 5’-ACAGCCTGATTACTGCCACT-3’
	R 5’-TCAGTTTGGTTCCCTCATCGGGTA-3’
ND6	F 5’-TTGGTTGTCTTGGGTTAGCA-3’
	R 5’-ACCAATCTCCCAAACCATCA-3’
Cytb	F 5’-CCCAACAGGATTAAACTCAGATGCAG-3’
	R 5’-GGATTGAGCGTAGAATGGCGTATG-3’
COX1	F 5’-ACGCCACTTCGCCATCATATTCGT-3’
	R 5’-ACATAGGTTGGTTCCTCGAATGTG-3’
ATPase6	F 5’-GGATTCCCAATCGTTGTAGCCA-3’
	R 5’-AAGGCCTAGGAGATTTGTTGATCC-3’
UCP2	F 5’-TCTGGATACCGCCAAGGT-3’
	R 5’-TTGTAGGCTGCGTGGA-3’
Tubulin	F 5’-CACGGTCATCGATGAAGTTCGC-3’
	R 5’-GCACTGGTCAGCCAGCTTGCGATTCC-3’
Actin	F 5’-GGTCATCACTATTGGCAACG-3’
	R 5’-ACGCATGTCAACGTCACACT-3’
PGC1α ChIP primers	L: 5’-CCAAAACAGGCAAATAGCAAAGATCC-3’
	R: 5’-GGCGGTTTTGTTGACTAAACATGG-3’

### Mitochondrial DNA isolation

Genomic DNA was extracted from 5-10mg of liver from mice according to manufacturer guidelines from the Puregene Core Kit A (Qiagen). 25ng of genomic DNA was used for real-time PCR to measure mitochondrial DNA using SensiMix SYBR and Fluorescein kit (Bioline Taunton, MA). All samples were assayed in duplicates and analyzed using a CFX96 Real-Time PCR Detection System (Bio-Rad, Hercules, CA). Mitochondrial encoded genes measured were ND4, ND6, Cytb, Cox1, ATPase6 and Actin as an internal control. Table 1 has a list of primers used.

### Mitochondria isolation

Livers were harvested from mice and washed with ice cold PBS. The livers were minced and homogenized in 10mL MSM-EDTA buffer (220mM Mannitol, 70mM Sucrose, 5mM MOPS, 2mM EDTA) on ice. Homogenized tissues were then centrifuged at 500xg for 10 minutes at 4°C to remove cell debris. The supernatants were centrifuged at 5000xg for 10 minutes (Thermo Fisher Sorvall RC 6+ with the SS-34 rotor) at 4°C to pellet mitochondria. Mitochondria were re-suspended in 5mL of ice cold MSM-EDTA and spun at 5,000xg for 10 minutes at 4°C. Supernatants were discarded and the mitochondria were re-suspended in 2.5mL of MSM-EDTA buffer and spun down at 5000xg for 10 minutes at 4°C. The supernatants were removed and mitochondria were re-suspended in 250mL MSM buffer with protease and phosphatase inhibitors (Roche, San Francisco, CA) and aliquoted in 50μL volumes and stored at -80°C.

### Mitochondria protein

Livers were harvested from mice and the weight was measured in grams (g). Mitochondria were isolated and protein was measured using the Lowry method [12].

### Mitochondrial Oxygen Consumption Oxygen Consumption in *STAT1* ^*+/+*^ and *STAT1* ^*-/-*^ liver mitochondria

Oxygen consumption was measured using a Clark-type oxygen electrode (Strathkelvin Instruments, North Lanarkhira, UK) at 30°C in respiration buffer (100mM KCl, 50mM MOPS, 1mM EGTA, 5mM KH2PO4, and 0.1% defatted BSA). Equal amounts of mitochondrial protein from *STAT1* ^*+/+*^ and *STAT1* ^*-/-*^ mice were used. The mitochondrial suspension was continuously stirred in the chambers. Oxygen consumption rates were measured in the presence of 5mM glutamate and 5mM malate to respiration as a donor to complex I and using 20mM succinate with 7.5μM rotenone as a a donor to complex II. State 3 was measured with 0.2mM ADP, and State 4 was analyzed following ADP-consumption as ADP limited respiration. The respiratory control ratio (RCR) was calculated as the ratio of 0.2mM stimulated state 3 to ADP-limited state 4 respiration. Following state 4 respiration, 2mM ADP was added to measure the maximal rate of ADP-stimulated respiration under the condition of saturating ADP followed by the addition of 0.2 mM dinitrophenol (DNP) to measure uncoupled respiration.

### Chromatin immunoprecipitation (ChIP) assay

*STAT1* ^*+/+*^ mice were either fed or fasted overnight and the livers were isolated,fixed with 1% formaldehyde for 10 min at 37°C, and then washed with ice-cold PBS containing 125mM glycine. Chromatin was sonicated and immunoprecipitated using specific antibodies exactly as described in the ChIP protocol provided by Upstate Inc. (Charlottesville, VA). The following antibodies were used: antisera polyclonal STAT1 and unspecific IgG antibody. The primers used in qPCR are listed in Table 1.

### Electron microscopy

Liver tissues were harvested from mice and washed in 1 x PBS and fixed in 2% paraformaldehyde, and 2% glutaraldedhyde in 0.1M cacodylate buffer. To determine the number of mitochondria per field thirty images were analyzed at 6000x magnification. Mitochondria were then counted in 30 fields.

### Western blot analysis

Equal amounts of protein were mixed with 2x loading buffer (2x Laemmli Sample Buffer (Bio-Rad) containing 1mM β-mercaptoethanol). Proteins were denatured at 95°C for 5 minutes and spun down at room temperature. Proteins were separated using SDSPAGE electrophoresis, and then transferred onto Immobilon-P polyvinyldifluoridine membrane (Millipore, Billerica, MA). Membranes were blocked for 1 hour at room temperature in 5% nonfat milk in TBS-TWEEN buffer (20nM Tris-HCl, pH 7.5, 500mM NaCl, and 0.05% Tween-20). Blots were incubated overnight at 4°C in primary antibody diluted in 5% BSA in TBS-TWEEN Buffer. After incubation, primary antibody was removed and the membranes are washed three times with 10mL TBS-TWEEN buffer for 9 minutes each. Blots were then incubated for 1 hour at room temperature with secondary antibody at 1:10,000 (mouse or rabbit) diluted in TBS-TWEEN. The blots were then washed again three times with 10mL TBS-TWEEN and developed using ECLPlus (Pierce Thermo Scientific, Rockford, IL).

The positive control for tyrosine 701 Phosphorylated STAT1 was obtained from pre-adipocytes treated with 2000U/mL of IFNβ for 30 minutes.

### White adipose tissue (WAT) cell size and count

Subcutaneous fat was isolated from six 12 week old *STAT1* ^*+/+*^ and *STAT1* ^*-/-*^ male mice. Cell size and count were analyzed by H&E staining. Four 20x images were taken from each mouse and analyzed using Image Pro Plus by Media Cybermetics.

### Statistics

Results are presented as the mean ± SEM. Statistical comparison was performed using two-tailed Student’s t-test or ANOVA where indicated. p-values less than 0.05 are considered statistically significant and annotated by *.

## Results

### *STAT1* ^*-/-*^ mice have enhanced energy expenditure

**Fig 1:**
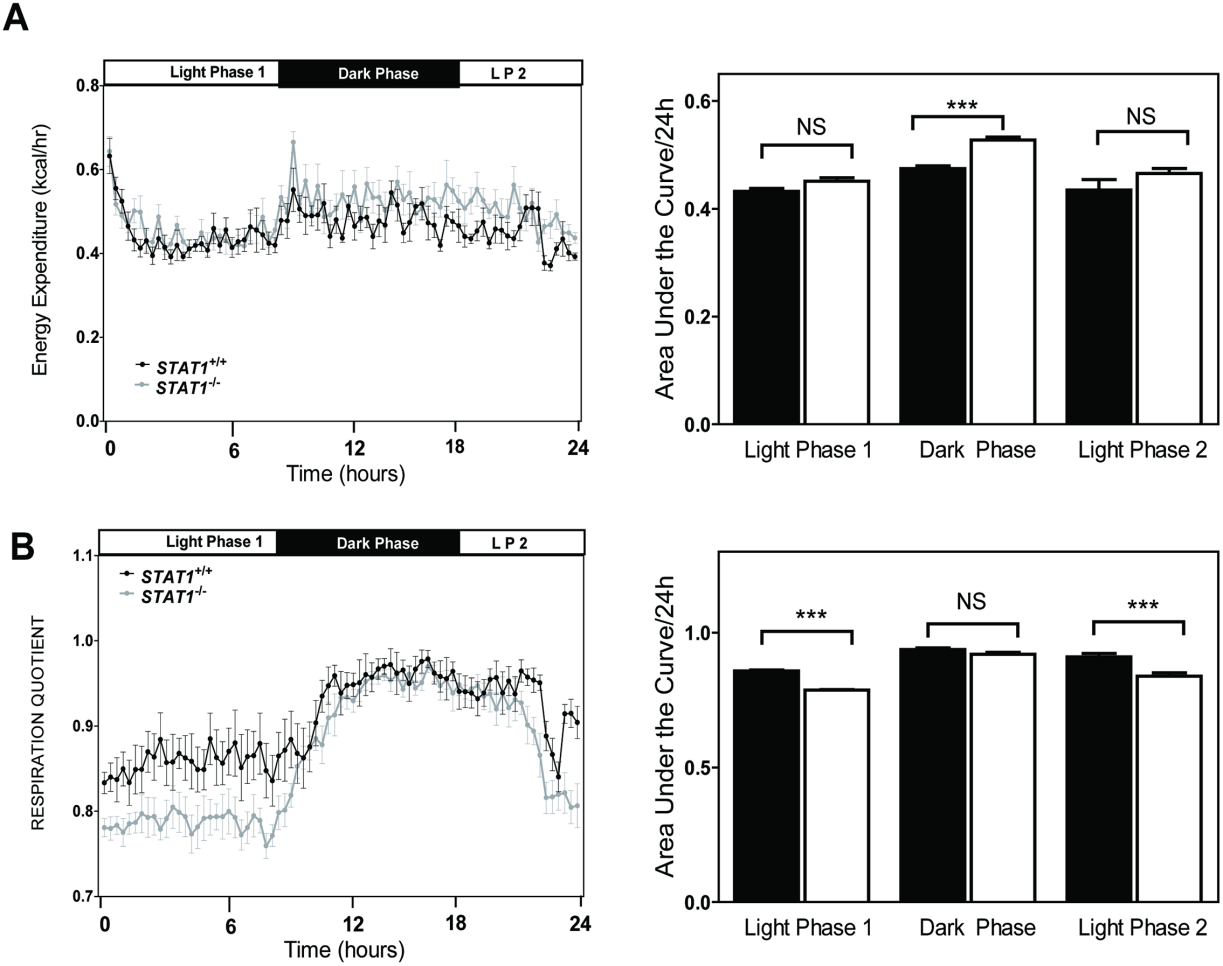
*STAT1* ^*-/-*^ mice display increased energy expenditure during starvation. The rates of a. energy expenditure (EE) and respiratory quotient (RER) were measured in mice over a 24 h period. The area under the circadian curves is shown to the right of the each figure. Values are the mean ± SEM, *** P<0.05 as determined by ANOVA with Bonferoni’s correction for multiple testing. b. Body composition of 24 h fasted *STAT1* ^*+/+*^ and *STAT1* ^*-/-*^ mice. Values are the mean ± SEM, * represents < 0.05. n = 6 mice per group.

We obtained a complete metabolic profile of 12-week-old *STAT1* ^*-/-*^ male mice, which showed that these mice had a 10% higher energy expenditure (Fig 1A) during the dark cycle, when mice are most active. In addition, *STAT1* ^*-/-*^ mice utilized more lipids during the light phase (LP1 and LP2) compared to *STAT1* ^*+/+*^ mice as measured by the respiratory quotient (Fig 1A). No changes in body weight (Fig 1B), food intake [*STAT1* ^*+/+*^, 2.82 ± 0.36 g/24 h (n = 5 per group) compared to *STAT1* ^*-/-*^, 2.97 ± 0.16 g/24 h (n = 6 per group)] or physical activity as measured on the treadmill [Maximum Speed *STAT1* ^*+/+*^ 19.33 ± 1.1 compared to *STAT1* ^*-/-*^ 19.00 ± 0.65 (m/min) and duration of run *STAT1* ^*+/+*^ 13.33 ± 1.1 min compared to *STAT1* ^*-/-*^ 12.83 ± 0.73 min] were detected between STAT1^+/+^ and STAT1^-/-^ mice (n = 6 mice per group). Although the treadmill endurance was not different between the *STAT1* ^*-/-*^ and *STAT1* ^*+/+*^ mice it is possible that the STAT1-/- mice have increased spontaneous activity in the dark phase. Although *STAT1* ^*-/-*^ mice displayed no changes in food intake, they showed increased body fat percentage and decreased lean mass weight (Fig 1B). The concentrations of various metabolites involved in glucose and fat metabolism were increased in the serum of fasted *STAT1* ^*-/-*^ mice, including lactate, ketone bodies, cholesterol, and glucose (Table 2). During fasting, fatty acids are oxidized to produce ketone bodies [13]. Increased ketone bodies in fed *STAT1* ^*-/-*^ animals suggested that they had increased fatty acid oxidation, which was consistent with the increased respiratory quotient (Fig 1A). Increased serum lactate suggests that entry of pyruvate into the TCA cycle may be impaired. Fatty acid oxidation increases the levels of acetyl CoA which is an allosteric inhibitor of pyruvate dehydrogenase complex. Thus the increased oxidation of fatty acids in *STAT1* ^*-/-*^ mice may have reduced entry of lactate into the TCA cycle. Although not statistically significant there is a suggestion of increased insulin in *STAT1* ^*-/-*^ mice (data not shown).

**Table 2:** Concentrations of serum metabolites in *STAT1* ^*+/+*^ and *STAT1* ^*-/-*^ mice.

	*STAT1* ^*+/+*^	*STAT1* ^*-/-*^
**Β-hydroxybutyrate (mg/dL)**	11.7 ± 1.1	15.12 ± 0.89 *
**Cholesterol (mg/dL)**	137.2 ± 6.4	110.8 ± 4.7 *
**Triglycerides (mg/dL)**	57.5 ± 4.4	54.2 ± 3.8
**Fatty Acids (nM)**	1.4 ± 0.12	1.4 ± 0.06
**L-Amino Acids (mg/dL)**	18.1 ± .035	18.3 ± 0.42
**Glucose (mg/dL)**	114.8 ± 9.2	150.8 ± 10.8 *
**Lactate (mg/dL)**	2.66 ± 0.25	3.6 ± 0.33 *

The serum metabolites were measured in fasted *STAT1* ^*+/+*^ and *STAT1* ^*-/-*^ male mice 8–10 week old. Values are the mean ± SEM,

* represents p < 0.05, Student’s T-test, n = 6 mice per group.

### *STAT1* ^*-/-*^ mice have defects in lipolysis

**Fig 2:**
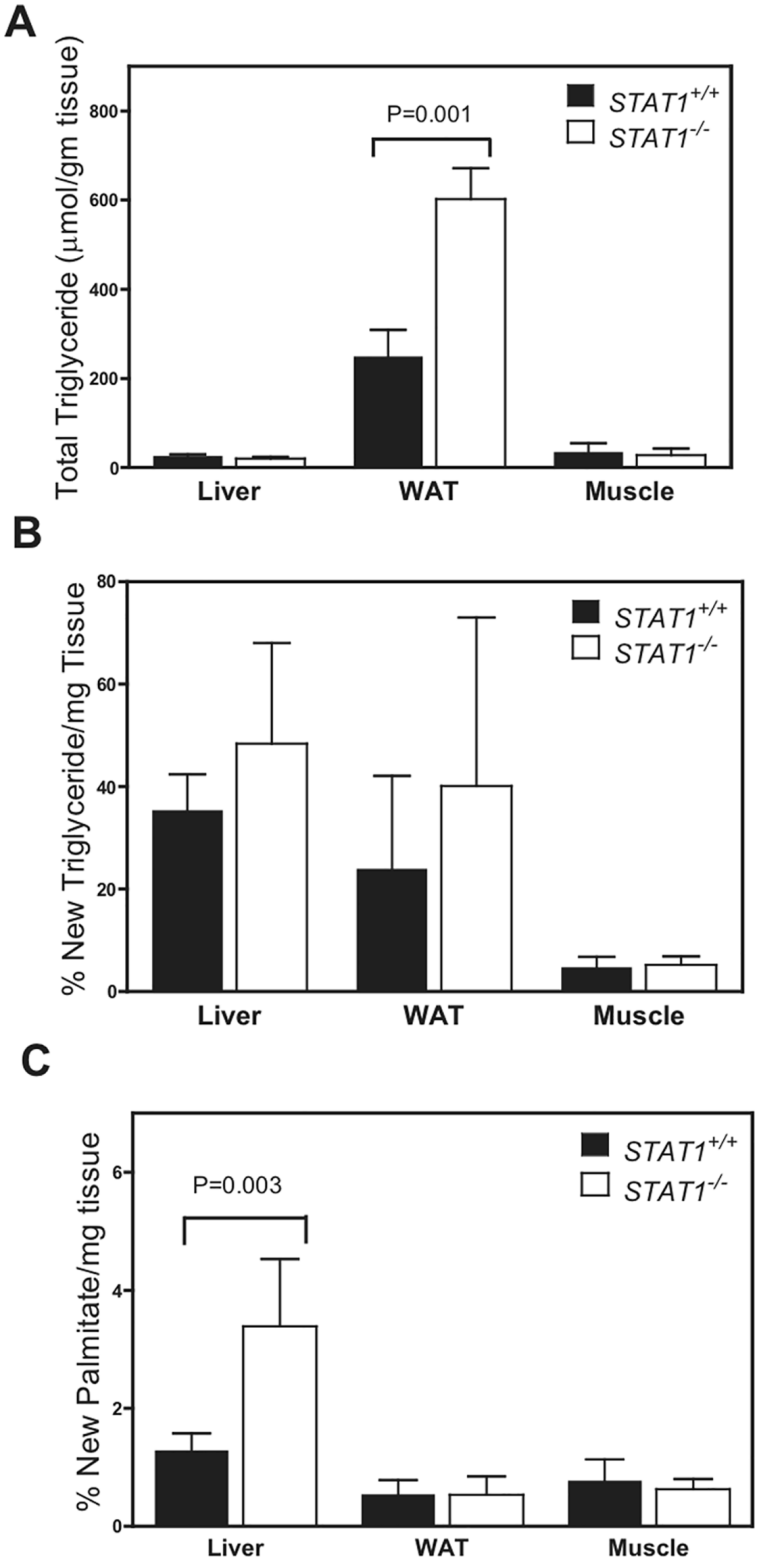
Total triglycerides are increased in *STAT1* ^*-/-*^ WAT. a. Total and b. newly synthesized triglycerides and c. the percent of the newly synthesized palmitate bound to triglyceride in tissues isolated from *STAT1* ^*+/+*^ and *STAT1* ^*-/-*^ mice that were treated with ^2^H_2_O. Values are the mean ± SEM, * presents p < 0.05 as measured by Student T-test (compared *STAT1* ^*+/+*^ liver tissue to *Stat1* ^*-/-*^ liver tissue, *STAT1* ^*+/+*^ white adipose tissue to *STAT1* ^*-/-*^ white adipose tissue and WT muscle to *STAT1* ^*-/-*^ muscle for n = 4 mice per group).

To further explore the change in fat distribution, triglyceride turnover was measured in mice administered deuterated water. There was no difference in rates of triglyceride synthesis in liver, muscle or white adipose tissue (WAT), but total amounts of triglycerides were increased in WAT of *STAT1* ^*-/-*^ mice (Fig 2A and 2B). There was also an increase in palmitate synthesis in the livers of *STAT1* ^*-/-*^mice (Fig 2C). This is consistent with a possible futile cycle in the liver where fatty acid oxidation is increased and fatty acid synthesis (palmitate) is also increased resulting in unaltered triglycerides.

**Fig 3:**
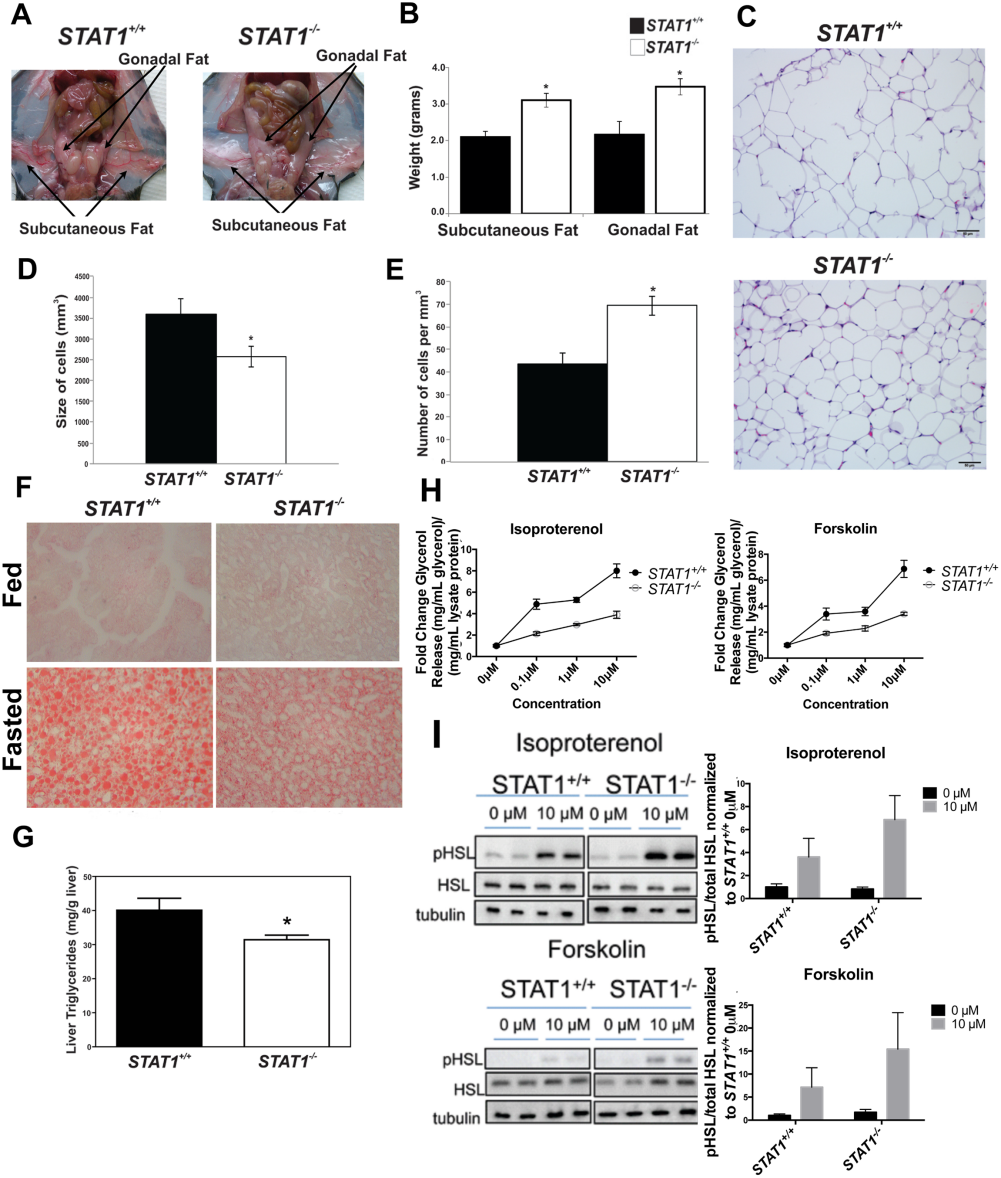
Fasted *STAT1* ^*-/-*^ mice have defective lipolysis of WAT. a. *STAT1* ^*+/+*^ (left panel) and *STAT1* ^*-/-*^ mice (right panel) were fasted and white adipose tissue depots were photographed. The images shown are representative of n = 5, 8–10 week old mice per group. b. Weight of subcutaneous and gonadal fat from *STAT1* ^*+/+*^ and *STAT1* ^*-/-*^ 24 h fasted mice. n = 4 8–10 week old male mice per group. c. H&E stains of subcutaneous fat from *STAT1* ^*+/+*^ and *STAT1* ^*-/-*^ mice were analyzed for d. size (in microns). The values represent mean size of cells (mm^3^) ± SEM for n = 6 mice per group. e. Quantification of the number of fat cells per mm^3^ from H&E stains of subcutaneous fat from *STAT1* ^*+/+*^ and *STAT1* ^*-/-*^ mice. * represents p < 0.05, Student’s T-test f. Oil Red O staining of liver from fed or fasted *STAT1* ^*+/+*^ and *STAT1* ^*-/-*^ mice. The images shown are representative of n = 4, 8–10 week old male mice per group. g. Liver triglyceride concentrations in *STAT1* ^*+/+*^ and *STAT1* ^*-/-*^ mice. The values represent mean ± SEM for n = 9 male 8–10 week old mice per group, * Significantly different p < 0.05, Student’s T-test. h. Glycerol release was measured in mature adipocytes that were isolated from subcutaneous fat and treated with various doses of isoproterenol or forskolin. n = 4, 12 week old male mice per group. The effect of genotype is significant at p<0.001; The genotype-concentration interaction is significant at p<0.05 for isoproterenol and at p<0.01 for forskolin. (**I**) Western blot and densitometric quantification of isoproterenol and forskolin induced phosphorylation of HSL normalized to total HSL.

The increase in triglycerides in *STAT1* ^*-/-*^ WAT prompted us to examine if there were abnormalities in the distribution of triglycerides and free fatty acids (FFA) in WAT or liver in *STAT1* ^*-/-*^ mice. There was little loss of subcutaneous or gonadal fat in fasted *STAT1* ^*-/-*^ mice compared with *STAT1* ^*+/+*^ mice (Fig 3A). The weight of subcutaneous and gonadal fat under fasted conditions confirmed the gross appearance of the fat pads in fasted *STAT1* ^*+/+*^and *STAT1* ^*-/-*^ mice (Fig 3B). Furthermore, white adipose cells in the subcutaneous fat were smaller and more numerous in *STAT1* ^*-/-*^ mice (Fig 3C, 3D and 3E). Increased energy expenditure is commonly associated with smaller adipocytes [14, 15]. We have not examined the role of STAT1 in adipocyte proliferation; however, it has been reported that STAT1 is increased during differentiation of WAT and may play a role in this process [16, 17]. It should be noted that changes in STAT1 expression were observed in *in vitro* differentiated 3T3L1 cells, not in primary white adipocytes.

Animals subjected to acute fasting accumulate lipid droplets in their livers as they utilize fat as their primary energy source. To examine if there were changes in fat droplets, liver sections were stained with Oil Red O, which stains neutral lipids. Although there were no gross differences between fasted or fed livers from *STAT1* ^*+/+*^ and *STAT1* ^*-/-*^ mice, Oil Red O staining showed that fasted *STAT1* ^*-/-*^ livers accumulated less lipid than *STAT1* ^*+/+*^ livers (Fig 3F). We measured the amount of hepatic triglycerides and determined that they were also decreased in *STAT1* ^*-/-*^ fasted livers (Fig 3G). These findings support the conclusion that under fasting conditions there is either increased fatty acid oxidation in the liver and/or decreased hydrolysis of triglycerides in WAT of *STAT1* ^*-/-*^ mice, resulting in the retention of triglycerides in adipose tissue.

One of the earliest events in induction of lipolysis is the activation of hormone sensitive lipase (HSL) and release of glycerol [18]. We measured the glycerol release in isolated adipocytes from *STAT1* ^*-/-*^ and *STAT1* ^*+/+*^ mice incubated with various doses of isoproterenol (ISO) (Fig 3h). Glycerol release was decreased in *STAT1* ^*-/-*^ white adipocytes treated with a variety of doses of ISO. There were no changes in basal release (Fig 3H). To interrogate whether there was a defect in coupling between the β-adrenergic receptors and activation of adenylate cyclase, we measured glycerol release in adipocytes treated with forskolin, which directly activates, adenylate cyclase (Fig 3H). Forskolin-stimulated glycerol release was also decreased in *STAT1* ^*-/-*^ adipocytes (Fig 3H). This result indicates that the defect in glycerol release is not due to a defect in the function of β-adrenergic receptors, but a decreased activity of adenylate cyclase or a defect downstream of adenylate cyclase in *STAT1* ^*-/-*^ adipocytes. If there was ineffective coupling of β-adrenergic receptors to adenylate cyclase, then forskolin activation of the enzyme would not be decreased in *STAT1* ^-/-^ adipocytes.

We also examined isoproterenol and forskolin-induced phosphorylation of HSL (Fig 3I). Phosphorylation of HSL is a marker for the relative activity of the enzyme. To our surprise, isoproterenol or forskolin-induced phosphorylation of HSL in *STAT1* ^*-/-*^ adipocytes was similar compared to *STAT1* ^*+/+*^ adipocytes. This suggests that the decreased glycerol release in isolated *STAT1* ^*-/-*^ adipocytes is downstream of HSL activation.

### Increased energy expenditure in mice is associated with increased mitochondrial biogenesis and uncoupling of the ETC

The elevated energy expenditure observed in *STAT1* ^*-/-*^ mice led us to examine if there were alterations in expression of the mRNA encoding PGC1α, an important marker of mitochondrial biogenesis [19]. PGC1α has a number of effects on cell metabolism including being a co-activator of peroxisome proliferator activated receptors (PPARs), and orchestrating the activity of transcription factors involved in mitochondrial proliferation, such as nuclear respiratory factors and estrogen-related receptors [20].

**Fig 4:**
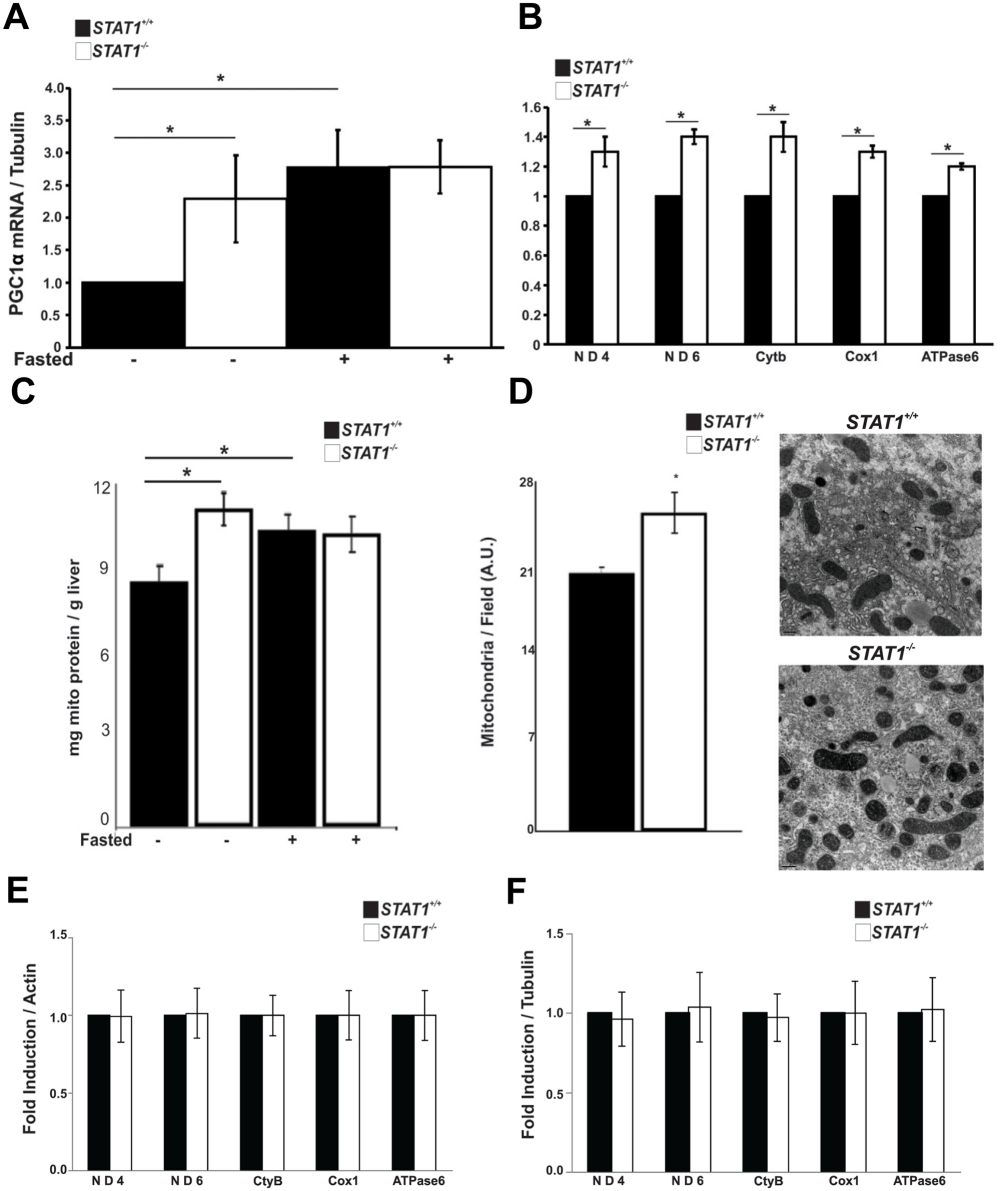
*STAT1* ^*-/-*^ mice display increased mitochondrial biogenesis in liver. a. Amounts of PGC1α mRNA were measured by qRT-PCR and quantitated relative to tubulin in livers of fed or fasted *STAT1* ^*+/+*^ and *STAT1* ^*-/-*^ mice. Values were normalized to *STAT1* ^*+/+*^ fed. * Significantly different p< 0.05(*STAT1* ^*+/+*^ fed vs. *STAT1* ^*+/+*^ fasted and *STAT1* ^*+/+*^ fed vs. *STAT1* ^*-/-*^ fed) as determined by two-way ANOVA + Holm-Sidak’s post-test, n = 5, 8–10 week old male mice per group. b. Mitochondrial DNA (ND4, ND6, Cytb, Cox1 and ATPase6) in livers of *STAT1* ^*+/+*^ and *STAT1* ^*-/-*^ mice was quantitated relative to actin DNA and normalized to *STAT1* ^*+/+*^. * Significantly different p< 0.05, Student’s T-test, correction for multiple testing c. Amount of mitochondrial protein in *STAT1* ^*+/+*^ and *STAT1* ^*-/-*^ livers, which was normalized to the weight of the liver. d. Number of mitochondria in *STAT1* ^*+/+*^ and *STAT1* ^*-/-*^ livers. n = 3 mice. * Significantly different p < 0.05, Student’s T-test. e. Mitochondrial DNA from *STAT1* ^*+/+*^ and *STAT1* ^*-/-*^ hearts. f. Amounts of mitochondrial RNAs in hearts in *STAT1* ^*+/+*^ and *STAT1* ^*-/-*^ mice. n = 6 mice per group.

Compared with *STAT1* ^*+/+*^ liver, PGC1α mRNA was increased in *STAT1* ^*-/-*^ liver suggesting there could be alterations in mitochondrial biogenesis in *STAT1* ^*-/-*^ livers (Fig 4A).

To explore this possibility, we measured the amount of mitochondrial DNA, protein and the total number of mitochondria per cell in the livers of *STAT1* ^*+/+*^ and *STAT1* ^*-/-*^ mice. Real-time PCR analysis indicated that the DNA content of mitochondrial genes encoding each complex of the electron transport chain was increased in livers of *STAT1* ^*-/-*^ mice (Fig 4B. In addition, the amount of protein from purified mitochondria per gram of liver was higher in *STAT1* ^*-/-*^ liver than in *STAT1* ^*+/+*^ livers and the number of mitochondria determined by electron microscopy (Fig 4C and 4D). Fasting tended to increase the amount of mitochondria protein in livers of *STAT1* ^*+/+*^ mice, but not in livers from *STAT1* ^*-/-*^ *mice* (Fig 4C). Changes in mitochondrial content were specific to the liver and not observed in heart, as seen in (Fig 4E and 4F).

**Fig 5:**
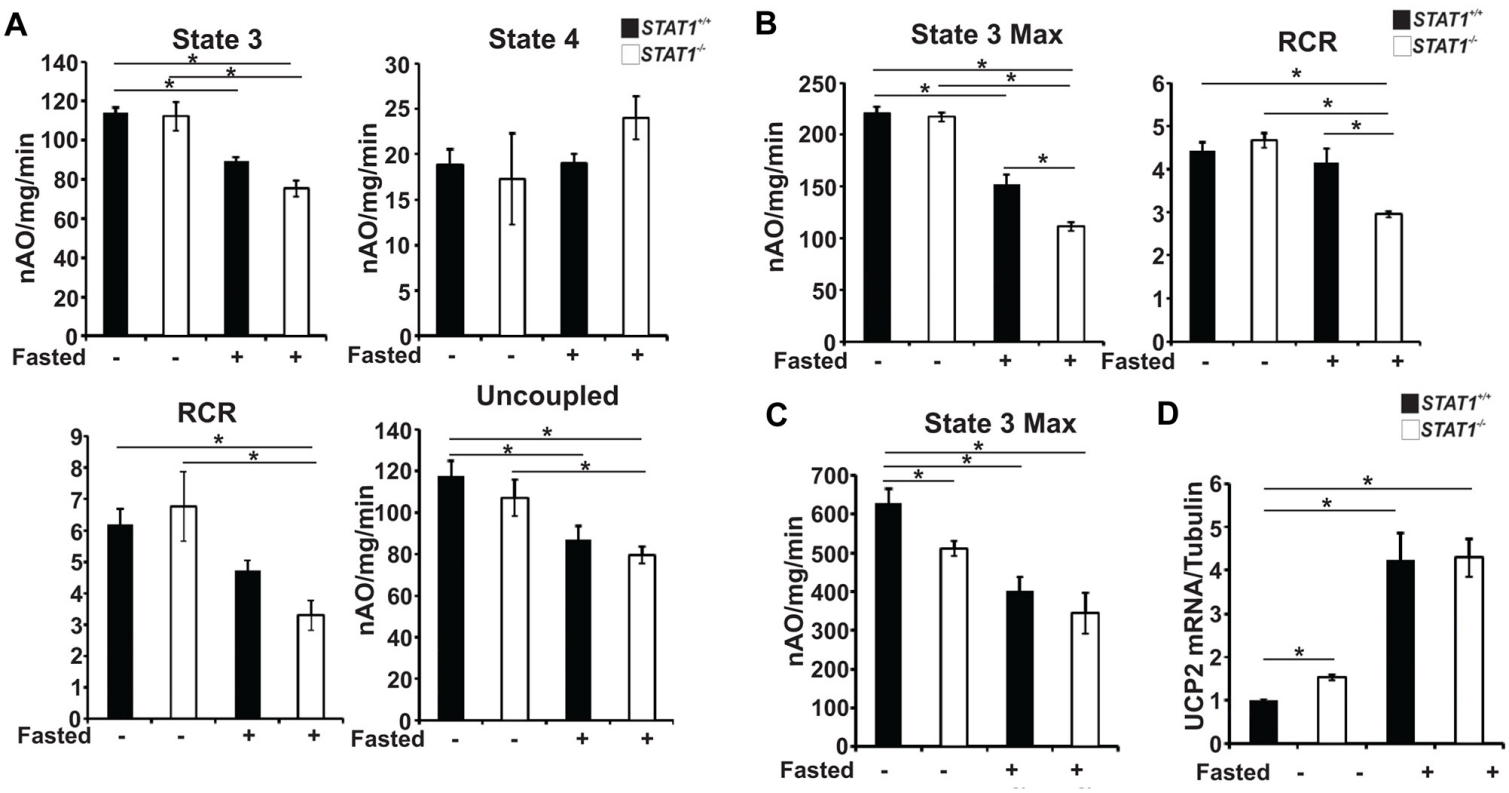
*STAT1* ^*-/-*^ mice have lower oxygen consumption. Oxygen consumption was measured from isolated liver mitochondria of male mice either fed or fasted using substrates that donate to specific sites in the ETC a. Glutamate-malate for Complex I, b. Succinate (+rotenone) for complex II, and c. TMPD-ascorbate (+rotenone) for Complex IV. Significance of p < 0.05 was determined with two-way ANOVA + Holm Sidak’s post test, n = 6, 8–10 week old male mice d. Amounts of UCP2 mRNA levels were measured in male mice either fed or fasted using qRT-PCR and quantitated relative to tubulin in livers. Values were normalized to *STAT1* ^*+/+*^ fed. Significance of p < 0.05 were determined by two-way ANOVA + Holm Sidak’s post test, n = 4, 8–10 week old male mice.

To determine the effect of STAT1 deletion on oxidative capacity, we measured oxygen consumption in liver mitochondria. Glutamate+malate was used to measure oxidative phosphorylation using a substrate that donates reducing equivalents to the ETC at complex I. There was a decrease in ADP-stimulated respiration with fasting in both *STAT1* ^*+/+*^ (*STAT1* ^*+/+*^ *ST*) and *STAT1* ^*-/-*^ (*STAT1* ^*-/-*^ *ST*) mitochondria (Fig 5A). The decrease with fasting was observed during ADP-stimulated state 3 respiration. To evaluate the role of STAT 1 knockout in the coupling of respiration, the respiratory control ratio (RCR; state 3/state 4) was assessed. The RCR decreased with fasting in both genotypes, much more prominent in *STAT1* ^*-/-*^ mice that exhibited an approximately 50% decrease. This decrease in coupling efficiency was due mostly to the decrease in ADP-stimulated state 3 respiration, which decreased significantly; accompanied by a modest trend toward an increasing state 4 respiration. The addition of the uncoupler dinitrophenol (DNP) did not correct the decreased oxygen consumption in *STAT1* ^*-/-*^ mitochondria, localizing the defect in respiration to the ETC rather than the phosphorylation apparatus (Fig 5A, Uncoupled). To further localize the site of the defect within the ETC, respiration using succinate (plus rotenone) as a substrate for complex II was measured (Fig 5B). The similar decreases in state 3 respiration for complex I and complex II substrates suggests that the ETC defect is located distal to complex I. To further localize the defect, ADP-stimulated respiration at complex IV was measured using N, N, N’, N’-tetramethyl-p-phenylenedamine (TMPD) as a substrate. TMPD donates electrons to complex IV via cytochrome c. Again, we found decreased ADP-stimulated respiration in *STAT* ^*-/-*^ mitochondria in both the fed and fasted states localizing a defect in intact mitochondrial to complex IV (Fig 5C).

To evaluate the mechanism of the decrease in RCR observed in *STAT1* ^*-/-*^ liver mitochondria following fasting, we measured UCP2, which is highly expressed in liver. We found (Fig 5D) that the mRNA expression of UCP2 in *STAT1* ^*-/-*^ livers in the fed state was elevated. Both *STAT1* ^*+/+*^ and *STAT1* ^*-/-*^ mice have increased UCP2 mRNA in fasting compared to the fed state. This may be due to increased UCP2 mRNA expression from fasting, as well as, differences in total mitochondrial protein. As stated above the *STAT1* ^*-/-*^ livers have more mitochondrial protein than *STAT1* ^*+/+*^ livers, which may compensate for differences in transcriptional expression of UCP2. Taken together, the data suggest that the increased energy expenditure in *STAT1* ^*-/-*^ mice during the dark phase may be due to uncoupling in the mitochondria by another mechanism besides UCP2, such as hydrophobic weak acids. Weak acids possess protonophoric activities through impermeable membranes as well as the increased number of mitochondria [21].

### STAT1 binding to the PGC1α promoter is inhibited by fasting

**Fig 6:**
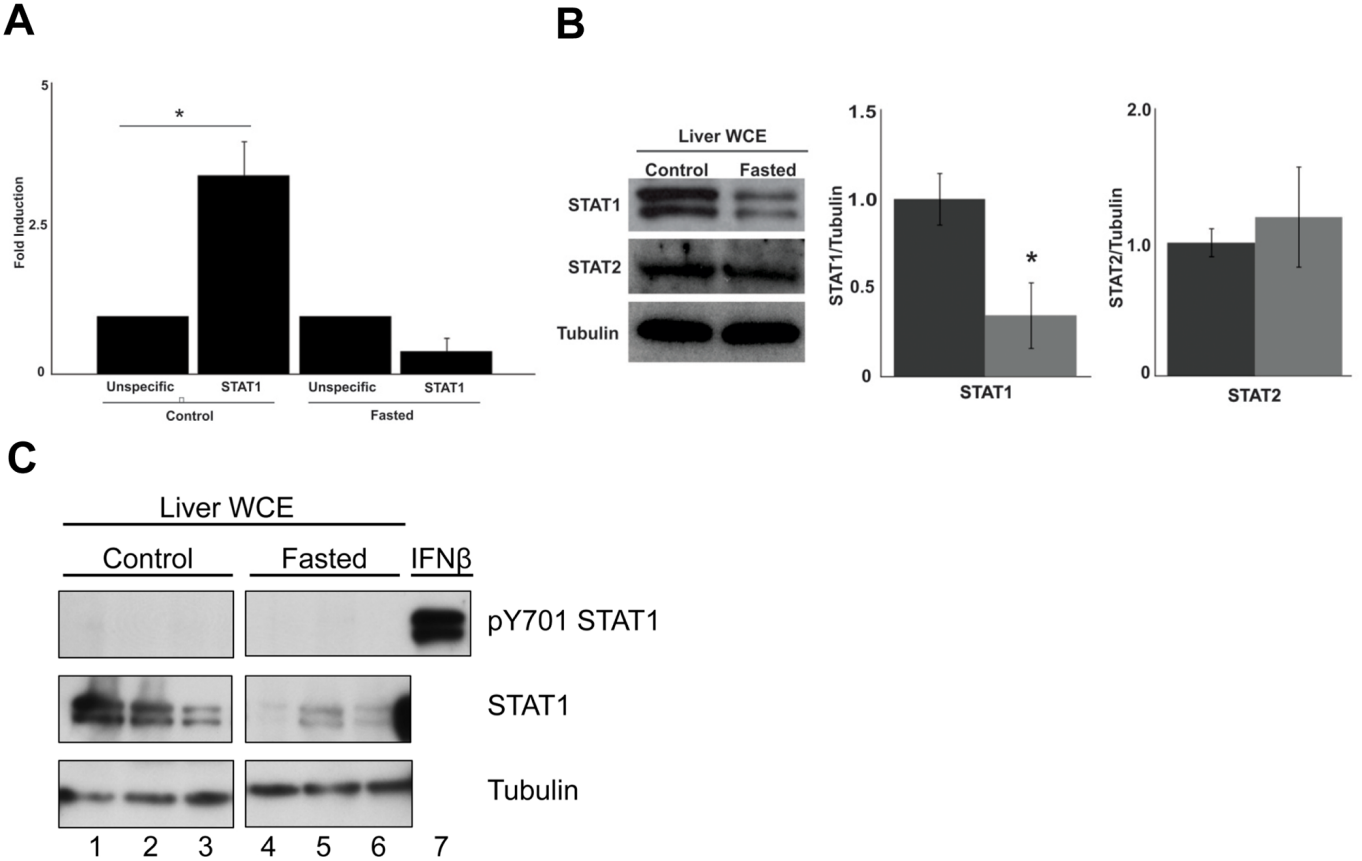
STAT1 binding to the PGC1α promoter is increased in fed mice, and levels of STAT1 protein are decreased in fasted mice. a. STAT1 binding to the PGC1α promoter was performed by ChIP assays from fed or fasted *STAT1* ^*+/+*^ mice. * Significantly different p < 0.05, Student’s T-test b. Whole cell liver extracts (WCE) from fasted or fed *STAT1* ^*+/+*^ mice were immunoblotted for STAT1, STAT2 and tubulin as a loading marker. STAT1 or STAT2 protein levels as determined by densitometry were quantitated relative to tubulin and then normalized to control. * Significantly different p < 0.05, Student’s T-test. c. STAT1-Tyrosine 701 phosphorylation in livers of fed and fasted *STAT1* ^*+/+*^ mice. Liver WCE from *STAT1* ^*+/+*^ and *STAT1* ^*-/-*^ mice under fed or fasted conditions were analyzed for total STAT1 (middle panel), STAT1 701 tyrosine phosphorylation (upper panel), or tubulin as a loading control (bottom panel). n = 3 mice per group.

PGC1α mRNA abundance was increased in *STAT1* ^*-/-*^ livers, suggesting that STAT1 represses the expression of PGC1α mRNA. To test this possibility, we performed chromatin immunoprecipitation (ChIP) assays using STAT1 antisera incubated with liver extracts from fed or fasted wild-type mice (Fig 6A). Under fed conditions STAT1 bound to the PGC1α promoter; however, there was no detectable binding of STAT1 with food deprivation.

These results are consistent with the hypothesis that under fed conditions, STAT1 represses mitochondrial biogenesis by inhibiting the expression of the nuclear genes activated by PGC1α. We did not detect tyrosine phosphorylated STAT1 in the livers of fed or fasted wild-type mice suggesting that the mechanism by which STAT1 repressed the expression of PGC1α did not involve cytokine activation of the JAK/STATpathway (Fig 6C). Interestingly, total STAT1 in the livers of fasted mice was decreased compared with fed animals (Fig 6B). The effects of food deprivation on STAT1 protein were selective in that STAT2 abundance in the liver was not altered by fasting. This result suggests that nutrient modulation of STAT1 protein expression may be by another mechanism by which food intake can modulate energy expenditure. It has been reported that STAT1 is also present in the mitochondria where in theory it might influence mitochondrial biogenesis [22]. The role of mitochondrial-localized STAT1 needs to be explored, but the actions of STAT1 on expression of PCG1α which is a nuclear-encoded gene are likely distinct from its direct effects on mitochondria function.

## Discussion

**Fig 7:**
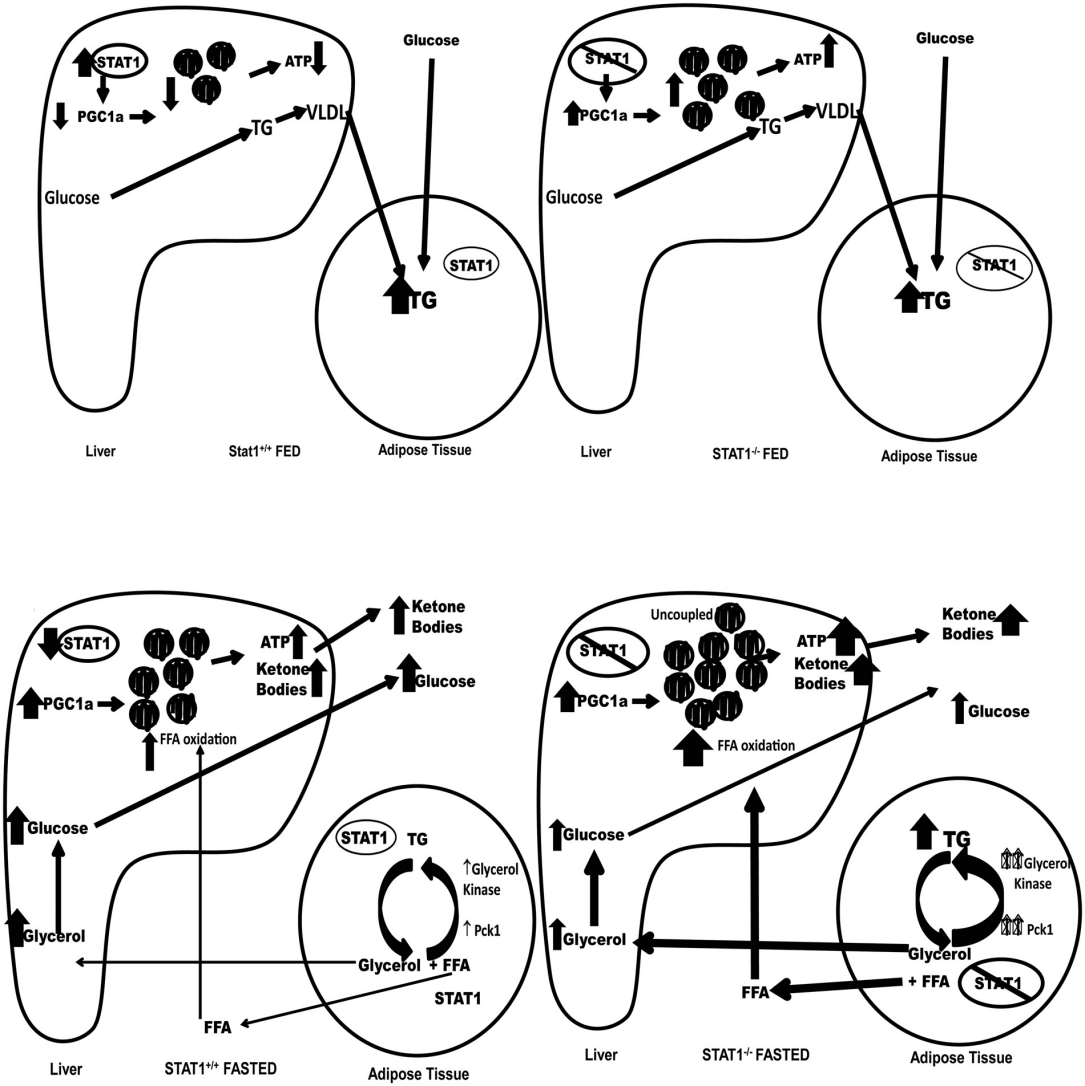
Proposed model for alterations in metabolism in *STAT1^-/-^* liver and adipose tissue.

The results of these studies indicate that in addition to its pivotal roles in preventing viral infections and inhibiting cell growth, STAT1 modulates lipid metabolism, energy expenditure and mitochondrial biogenesis. *STAT1* ^*-/-*^ mice display increased energy expenditure in the absence of increased food intake or differences in weight (Fig 1). We propose that STAT1 modulates oxidative phosphorylation and loss of STAT1 results in uncoupling of the ETC (Fig 5). It is possible that enhanced energy expenditure in *STAT1* ^*-/-*^ mice may alter amounts of activity of brown fat. We did not observe overt differences in brown fat function or quantities in *STAT1* ^*-/-*^ mice. A model summarizing our findings is shown in Fig 7.

In STAT1^+/+^ fed (upper panels), glucose is used to make triglycerides which is packaged into VLDL in the liver. VLDL transports lipids to adipose tissue for storage. In the adipose tissue glucose will be used to synthesize new triglcyerides. In STAT1^-/-^ livers (upper right panel), the number of mitochondria is increased as well as the production of ATP. These mice generate VLDL from glucose and transport the lipids to the adipose tissue. However, the STAT1^-/-^ adipose tissue also have increased re-esterification of triglycerides as well as generation of new triglycerides from glucose resulting in increased lipid stores.

In fasted *STAT1* ^*+/+*^ mice, the adipose tissue undergoes lipolysis with the activation of hormone sensitive lipase (HSL-P). Triglyceride is hydrolyzed to glycerol and fatty acids. The glycerol is transported to the liver and used for gluconeogenesis. The glucose is released from the hepatocyte and used by other tissues (brain, RBS, muscle). The fatty acids from adipose tissue lipolysis are transported to the liver and enter the mitochondria for β-oxidation. The end product, acetyl CoA is used for ketone body synthesis. In *STAT1* ^*-/-*^ mice very little glycerol is released during lipolysis because most of the glycerol is re-esterfied with FFA back to triglycerides in adipose tissue due to increased phosphoenolpyruvate carboxykinase (Pck1) and increased glycerol kinase expression data not shown). The *STAT1* ^*-/-*^mice have more mitochondria which results in greater fatty acid oxidation and ketone body synthesis.

Increased energy expenditure in *STAT1* ^*-/-*^ mice accompanies increased mitochondrial biogenesis. One mechanism by which STAT1 inhibits mitochondrial biogenesis is by suppressing the expression of PGC1α. ChIP assays using livers from fed mice demonstrate binding of STAT1 to the promoter of PGC1α. In contrast, there was no binding detected of STAT1 to the promoter in fasted animals (Fig 6A). Although there is a weak GAS site 177bp upstream of the transcriptional start site in the PGC1α promoter, we are not able to determine if this site is physiologically relevant since STAT1 regulation of PGC1α has not been observed in cell culture models. Therefore we cannot examine whether deletion of this GAS site affects STAT1 binding. Alternatively, STAT1 may not directly bind DNA, but interacts with another transcription factor such as calcium/calmodulin kinase IV or MEF2 both of which regulate PGC1α transcription [23].

Cytokine-mediated activation of STAT1 under most scenarios requires the protein to be tyrosine phosphorylated to induce transcription. However, there are genes whose activation does not require STAT1 to be phosphorylated [2]. We have not detected tyrosine phosphorylated STAT1 in the livers of either fasted or fed wild-type mice, suggesting that cytokine activation of the classic JAK/STAT pathway is not involved in repressing PGC1α (Fig 6C).

It remains to be determined whether the actions of STAT1 on energy expenditure in mice are interrelated and coordinated with its actions on the breakdown of triglycerides. It is paradoxical that while *STAT1* ^*-/-*^ mice show increased energy expenditure, they have higher fat mass in WAT and decreased β-adrenergic stimulated lipolysis. Forskolin-stimulated glycerol release which directly activates adenylate cyclase was also decreased in *STAT1* ^*-/-*^ mice suggesting that β-adrenergic receptor coupling is not defective in *STAT1* ^*-/-*^ adipocytes (Fig 3H). Surprisingly, the levels of phosphorylated HSL were not significantly different in both isoproterenol and forskolin-treated *STAT1* ^*-/-*^ compared with *STAT1* ^*+/+*^ adipocytes (Fig 3i). There are a variety of mechanisms, which could account for the discrepancy between decreased glycerol release, and normal ISO and forskolin-induced phosphorylation of HSL. One possible explanation for this phenomenon is the existence of a futile cycle where reduced glycerol release (lack of lipolysis) in *STAT1* ^*-/-*^ WAT, leads to the re-esterification of glycerol to triglycerides and retention of lipid under fasting conditions [24]. Activation of this futile cycle could be initiated because of a defect in glycerol transport or the activity of the lipases beside HSL needed to release glycerol. Monoglyceride (MAG) lipase hydrolizes monoglycerides but not diglycerides or triglycerides. A decrease in its activity could result in decreased release of glycerol resulting in MAG being re-esterified back to triglycerides and maintaining the higher fat mass. Decreased release of FFA from abdominal fat likely stimulates mitochondrial biogenesis in the liver to increase ATP production. The re-esterification of FFAs in fasted *STAT1* ^*-/-*^ WAT alters lipid metabolism in the liver. The liver compensates for the lack of FFAs released from the WAT by synthesizing more palmitate in the liver. In the *STAT1* ^*-/-*^ livers, the newly synthesized FFAs undergo β-oxidation to generate ATP and thus create a futile cycle. This futile cycle prevents lipid accumulation in the livers of fasted *STAT1* ^*-/-*^ mice (Fig 3).

Complementing our findings of a role of STAT1 in energy metabolism, mitochondria are also involved in a variety of cellular functions which are influenced by STAT1 such as cell cycle regulation, oxygen sensing [25, 26], and antiviral defense [27]. The observation that STAT1 modulates mitochondrial biogenesis is consistent with the actions of this transcription factor as an essential component of innate and adaptive immune responses, and growth regulation, all of which impinge on ATP production. It is becoming evident that there is cross talk between the mitochondria and the nucleus that coordinate responses to changes in homeostasis [28]. Although we have defined a role of STAT1 in regulation of mitochondria biogenesis, the actions of this transcription factor in regulation of mitochondrial function will also likely be important to pathogen and cell survival-mediated stresses, the primary targets of STAT1. The actions of STAT1 in response to food deprivation appear to be liver-specific. However, it is possible that different stresses will dictate which tissue is sensitive to STAT1-mediated changes in mitochondria biogenesis. Since our studies were performed on mice where there is a global knock-out of STAT1, it is possible that the effects we observed in liver are secondary to disrupted expression of the protein in other tissues. Examination of this possibility will require the use of floxed STAT1 animals and a STAT1 transgene which is selectively expressed in liver. However, both of these approaches have limitations. Now that a connection has been identified between STAT1, energy homeostasis, triglyceride turnover, and mitochondria biogenesis, the challenge will be to define the molecular details of this pathway and determine its role in other STAT1-mediated events such as immunity and cell growth.
